# Neonatal Perforated Appendicitis Attributed to Localized Necrotizing Enterocolitis of the Appendix: A Review

**DOI:** 10.21699/jns.v6i3.535

**Published:** 2017-08-10

**Authors:** Andrew Tumen, Pranit N. Chotai, John Matthew Williams, Adrianne Myers-Webb, Ramesh Krishnan, James W Eubanks III

**Affiliations:** 1 Division of Pediatric Surgery, Department of Surgery, University of Tennessee Health Science Center, Le Bonheur Children’s Hospital, Memphis, TN, USA.; 2 Department of Surgery, Vanderbilt University Medical Center, Nashville, TN, USA.; 3 Division of Neonatology, Department of Pediatrics, Regional One Health, University of Tennessee Health Science Center, Memphis, TN, USA.

**Keywords:** Neonatal perforated appendicitis, neonatal appendicitis, necrotizing enterocolitis, localized NEC.

## Abstract

Neonatal appendicitis is a rare clinical entity associated with remarkable morbidity and mortality. Appendicular perforation is common and the diagnosis is usually made intra-operatively. The causative etiology of neonatal perforated appendicitis (NPA) is a subject of debate and has not been elucidated. Although many etiologic theories exist, increasing evidence suggests a subset of NPA cases may represent a form of necrotizing enterocolitis (NEC) localized to the appendix. We herein present a review of the current literature to include cases of NPA attributed to localized NEC. A high index of clinical suspicion and early laparotomy are recommended.

**Introduction**

Neonatal appendicitis is exceedingly rare with an incidence of approximately 40 per 100,000 live births [1,2]. This condition is fatal in approximately a quarter of cases, which can be attributed to its predilection in preterm infants, diagnostic delay, high rate of appendicular perforation, and associated comorbidities [2-4]. The causative etiology remains uncertain, and may not be related to intraluminal obstruction as in older patients [4-7]. NEC is a commonly encountered gastrointestinal emergency in the neonatal intensive care unit (NICU) with a reported incidence of 1-7.7% admissions [8]. Neonatal perforated appendicitis (NPA) is uncommon and increasingly being recognized as a localized form of NEC involving the appendix [4,7,9,10]. Notably, NEC is the most common preoperative mis-diagnosis for NPA confirmed intra-operatively [8]. Here we present a review of current literature involving a subset of reported NPA cases where localized NEC of the appendix was thought to be a causative etiology. The purpose of this review is to better understand the clinical presentation, diagnosis, and management of this uncommon etiology of the neonatal acute abdomen.

**Etiology / Pathophysiology**

The rarity of neonatal appendicitis has been attributed to various protective features of the neonatal cecal appendage including a funnel-shaped anatomy, infrequency of lymphadenitis-causing infections, as well as soft diet, and recumbent posture [4,5,7]. These characteristics are thought to render the neonatal appendix less prone to intraluminal obstruction than their older counterparts [4,5,7]. Three etiological theories of neonatal appendicitis exist and are supported by the incidence of associated conditions [4]: impaired immunity such as prematurity (arguing it as a variant of NEC) [7,9,10-12]; vascular insufficiency and hypoxemia (cases associated with cardiorespiratory failure) [9,13]; and finally, intestinal obstruction such as Hirschsprung’s disease or strangulated Amyand’s hernia [7,14,15]. Interestingly, cases attributed to inguinal hernia have better outcomes due to discrete physical findings and early surgical exploration [2,5,14].

NEC usually presents in preterm neonates during the second week of life, after initiation of enteral nutrition, although the age of onset varies inversely with gestational age [16]. Predisposing factors include respiratory distress, congenital heart disease, low birth weight, IUGR, formula feeding, and intestinal dysbiosis, among others [10,16]. While fulminant NEC usually involves the colon and/or small bowel globally, perforated appendicitis may be the result of NEC confined, at least initially, to the appendix [7,9-12]. This NEC appendicopathy may be either a local pathology or part of a more diffuse disease process [6,17]. Primary appendicitis can be difficult to differentiate from NEC confined to the appendix. Furthermore, isolated NEC appendicopathy and transmural appendicitis cannot be histologically distinguished [6,7,18].

**Clinical Features**

The clinical presentation of appendicitis in the newborn is nonspecific and overlaps with that of NEC [3,4,9,11]. The most common findings are abdominal distension, tenderness, feeding intolerance, and fever [2,3,17]. Approximately 50% of cases occur in premature neonates and a third of cases are initially diagnosed as NEC [3]. Historically, the mortality rate was nearly 80% and the diagnosis was often made on autopsy [2,7]. The reported mortality rate of NPA in recent years ranges from 18-28% [2,3]. Neonates with appendicitis are now usually brought to the operating room emergently, with a provisional diagnosis of NEC, and diagnosed intra-operatively [3]. Indeed, there are only 3 reported cases where appendicitis was diagnosed preoperatively [2,3]. Diagnostic delay may lead to unrecognized perforation and rapid development of abdominal sepsis, more so in the premature infant due to compromised integrity of the bowel wall and omentum [5]. In a large retrospective series by Raveenthiran et al., appendicular perforation was noted on laparotomy in 44 of 52 cases of intra-abdominal neonatal appendicitis [3]. In that series, pneumoperitoneum on plain abdominal radiography was present in only 52% of perforated cases [3]. Paradoxically, perforation heralds significantly lower mortality than non-perforated cases due to timely clinical recognition, highlighting the benefit of early surgical intervention [3].

**Diagnostic Evaluation**

Clinical diagnosis of NPA is challenging due to its non-specific presentation, as well as a limited clinical history and physical examination in neonates. Furthermore, vital signs and traditional laboratory studies may not accurately reflect illness severity in preterm neonates. Regardless, serial abdominal examinations relying on the diagnostic acumen of the surgeon and ICU monitoring with frequent laboratory assessment remain critical components of patient evaluation and work-up. Notably, no clinical or radiologic criteria are currently available to distinguish NPA vs NEC prior to surgical exploration. Supine, upright, and lateral abdominal radiographs are often the initial imaging study obtained in suspected NEC, however they commonly fail to detect intra-peritoneal free air in the setting of NPA [3]. Graded compression ultrasonography is highly specific for detecting appendicitis in children; however, sensitivity varies greatly and is operator dependent [19]. Computed tomography (CT) scans of the abdomen should not be obtained routinely in neonates due to poor quality and the risk of unnecessary radiation exposure [20,21]. As such, many authors recommend the use of ultrasound as an initial imaging modality in infants and children undergoing diagnostic imaging for suspected appendicitis [17,20].

**Management**

Given the rarity of NPA, management is currently driven by anecdotal experience and isolated case reports. Classic NEC in a stable neonate has been shown to be successfully managed conservatively with bowel rest, decompression, and empiric antibiotics, with or without peritoneal drainage [22-24]. Recent trials have suggested that initial treatment with either peritoneal drainage or laparotomy may have equivalent results in stage IIIB NEC [23,24]. In contrast, non-operative management of NPA is usually not recommended [1]. The reported failure rate for non-operative management of perforated appendicitis in children ranges from 10% to 40% [25-27]. In light of increasing non-operative management of NEC, a missed diagnosis of perforated appendicitis may explain failure of drain placement in a subset of patients [1]. Patients who fail conservative management may be at higher risk for postoperative complications and extensive bowel resection [25,28,29]. Given their frailty and limited reserve, preterm neonate, who constitute a substantial portion of patients with NPA, may not tolerate the infectious sequelae of such complications. Thus, timely differentiation of NPA from non-appendicular causes of abdominal pain is of critical importance. However, given the lack of distinguishing diagnostic criteria and the necessity of prompt intervention, an early surgical intervention may be prudent in a neonate with suspected appendicular pathology.

Our review of the extant literature identified a total of 95 originally reported cases of NPA. Most cases were associated with concomitant morbidities, the most common being inguinal hernia, Hirschsprung’s disease, and cardiorespiratory failure. Ten cases were without systemic comorbidities and attributed to a localized NEC involving the appendix (Figure 1A,1B). The majority of these neonates were premature (8/10), and all had low birth weight. Abdominal distension was universally present on initial examination. Five cases presented with abdominal manifestation in the first week, and nine of the ten patients suffered abrupt clinical decline prompting surgical intervention in the second week of life. Half of these patients displayed pneumatosis intestinalis and six patients demonstrated pneumoperitoneum on plain radiograph. Abdominal wall erythema was noted in three, a RLQ mass was palpated in four, and blood was found in the stool of four of these neonates. Seven of these ten cases were initially diagnosed as NEC preoperatively. Two others were misdiagnosed as Hirschsprung’s disease and bowel obstruction, respectively, before perforated appendicitis was identified on laparotomy. The presence of right-sided intramural gas and pneumoperitoneum in an otherwise healthy premature neonate without comorbid risk factors for appendicitis may indicate that a localized variant of NEC is responsible for appendicular perforation.

**Conclusion**

NPA is uncommon; however, with improving survival of newborns with associated comorbidities, more NPA cases can be anticipated in surgical practice [4]. A similar causality has been implicated in the rising incidence of NEC [4]. The ambiguity of whether NPA represents a unique clinical entity or a manifestation of localized NEC necessitates further investigation of the relationship between NPA and NEC. Nonetheless, NPA should be considered early in the neonate with an acute abdomen. Routine CT scanning is not encouraged in neonates and future clinical research is needed to better understand the role of ultrasonography in preoperative diagnosis. Awareness of the multifactorial etiologic possibilities, nonspecific presentation, and limitations of conventional imaging modalities is necessary in improving outcomes of NPA. A low threshold for early surgical intervention may help mitigate the high morbidity and mortality associated with perforated appendicitis in a premature neonate presenting with pneumatosis intestinalis and/or intra-peritoneal, extra-luminal free air. 

**Figure:**
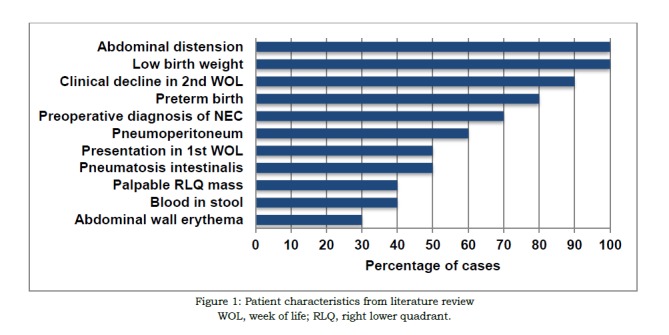
Figure 1: Patient characteristics from literature review

**Figure:**
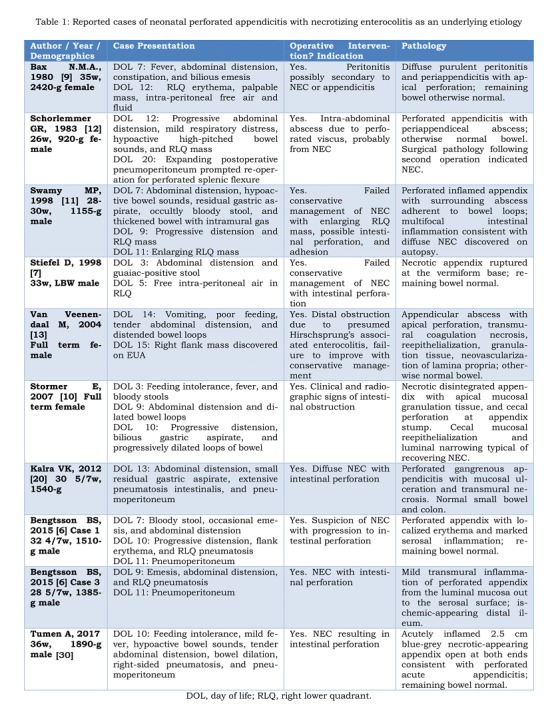
Table 1: Reported cases of neonatal perforated appendicitis with necrotizing enterocolitis as an underlying etiology

## Footnotes

**Source of Support:** None

**Conflict of Interest:** None
